# Three-dimensional reconstruction of coronary arteries and plaque morphology using CT angiography – comparison and registration with IVUS

**DOI:** 10.1186/s12880-016-0111-6

**Published:** 2016-01-19

**Authors:** Lambros Athanasiou, George Rigas, Antonis I. Sakellarios, Themis P. Exarchos, Panagiotis K. Siogkas, Christos V. Bourantas, Hector M. Garcia-Garcia, Pedro A. Lemos, Breno A. Falcao, Lampros K. Michalis, Oberdan Parodi, Federico Vozzi, Dimitrios I. Fotiadis

**Affiliations:** Unit of Medical Technology and Intelligent Information Systems, Department of Materials Science and Engineering, University of Ioannina, PO Box 1186, GR 45110 Ioannina, Greece; FORTH-Institute of Molecular Biology and Biotechnology, Department of Biomedical Research, GR 45110 Ioannina, Greece; Department of Cardiology, Barts Heart Centre, London, UK; Department of Cardiovascular Science, University College London, London, UK; Department of Interventional Cardiology, Erasmus University Medical Centre, Thoraxcenter, Rotterdam, The Netherlands; Department of Interventional Cardiology, Heart Institute, University of São Paulo, Medical School, São Paulo, Brazil; Michaelidion Cardiac Center and Department of Cardiology, Medical School, University of Ioannina, GR 45110 Ioannina, Greece; Institute of Clinical Physiology, National Research Council, Pisa, IT 56124 Italy

**Keywords:** Computed Tomography Angiography, Intravascular ultrasound, Plaque characterization, 3D reconstruction

## Abstract

**Background:**

The aim of this study is to present a new methodology for three-dimensional (3D) reconstruction of coronary arteries and plaque morphology using Computed Tomography Angiography (CTA).

**Methods:**

The methodology is summarized in six stages: 1) pre-processing of the initial raw images, 2) rough estimation of the lumen and outer vessel wall borders and approximation of the vessel’s centerline, 3) manual adaptation of plaque parameters, 4) accurate extraction of the luminal centerline, 5) detection of the lumen - outer vessel wall borders and calcium plaque region, and 6) finally 3D surface construction.

**Results:**

The methodology was compared to the estimations of a recently presented Intravascular Ultrasound (IVUS) plaque characterization method. The correlation coefficients for calcium volume, surface area, length and angle vessel were 0.79, 0.86, 0.95 and 0.88, respectively. Additionally, when comparing the inner and outer vessel wall volumes of the reconstructed arteries produced by IVUS and CTA the observed correlation was 0.87 and 0.83, respectively.

**Conclusions:**

The results indicated that the proposed methodology is fast and accurate and thus it is likely in the future to have applications in research and clinical arena.

## Background

Accurate detection and quantification of coronary plaque burden and assessment of its characteristics affect prognosis in patients with coronary artery disease and they are important for assessing the effect of new pharmacological treatments [1–3]. Intravascular ultrasound (IVUS) is considered as the method of choice in assessing atherosclerotic plaque burden [4] and plaque characteristics [5]. However, IVUS is an invasive method which has risks, it cannot be used in the contemporary practice for the study of the entire arterial tree and it is not indicated for asymptomatic individuals.

Coronary Computed Tomography angiography (CTA) [6] is a non-invasive imaging modality able to visualize the coronary arteries. Based on the Hounsfield Units (HU) scale, the inner and outer wall of the vessels can be estimated and the atherosclerotic plaques can be classified to Calcified (CP) and Non-Calcified (NCP) [7]. Several studies [8, 9] have demonstrated that CTA derived metrics such as the luminal dimensions, plaque burden and the composition of the plaque can provide useful prognostic information [10], while Papadopoulou et al. [11] demonstrated that CTA has a value in assessing changes in the composition of the plaque in patients admitted with an acute coronary event. Moreover, an accurate 3D reconstruction of the coronary arteries can provide models that allow comprehensive visualization of the vessel geometry and assessment of the distribution of different plaque types in space and permit blood flow simulation and evaluation of the role of the local hemodynamic forces on plaque progression [12].

Several methods have been presented in the literature to assess plaque burden and characterize the composition of the plaque in CTA [13, 14]. The outcome of the proposed approaches was compared with the estimations of IVUS which was treated as the reference standard. Leber et al. [15] manually detected CP in both CTA and IVUS and compared their volume. Fiduciary points such as: side branches, calcified plaques and the origin of the stents were manually selected to identify matching between CTA and IVUS. Following the same rationale Voros et al. [16] studied the correlation between plaques detected by IVUS and CTA and compared both their volume and areas. In a similar attempt Brodoefel et al. [17] compared the CP volume estimated by Virtual Histology (VH) and a software (SUPERPlaque software) developed to characterize the composition of the plaque in CTA. Recently Graaf et al. [18] attempted to correlate the plaque volumes estimated by a plaque characterization CTA software (QAngio CT 1.1, Medis medical imaging systems) and VH-IVUS. However, all the above mentioned methods required manual interaction for the extraction of the lumen and outer vessel wall borders and did not present any plaque detection or 3D plaque reconstruction method. They only correlated the plaques using manual estimations [8, 15, 16, 19] or commercially available software [17, 18, 20]. They focus only on volume comparison ignoring the spatial location of the volumes within the studied IVUS and CTA segments.

In this work, we present a new-semi automated methodology for the 3D Reconstruction of coronary arteries and plaque morphology using CTA. In order to facilitate the accuracy of the 3D plaques detected by our method we used as reference standard the results of a recently presented IVUS plaque characterization method [21]. The detected 3D CP plaques were extensively compared to IVUS plaques and a significant correlation was observed between CTA and IVUS. Additionally, the reconstructed inner and outer vessel wall models derived by the proposed methodology were compared with those reconstructed using the IVUS borders. The comparison results demonstrated an excellent agreement. The innovative aspects of the proposed methodology are:

- it focuses on the reconstruction of both the lumen, outer vessel wall and the CP plaques,
- the methodology is semi-automated and the user intervenes only in the plaque parameters adaptation stage (third stage),
- it is able to reconstruct the lumen, outer vessel wall and plaque geometry taking into consideration the vessel curvature and thus it allows assessment of the distribution of the plaque in the 3D space,
- a comparison is performed in 3D space by evaluating the volumes of the lumen, outer vessel wall and CA, derived by CTA and by IVUS after a spatial co-registration of the IVUS onto the CTA data.

## Methods

**Fig. 1:**
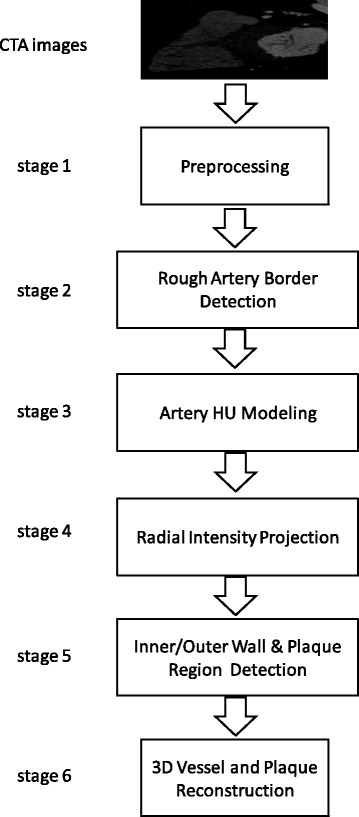
The stages of the proposed methodology starting from the initial CTA images and concluding with a full 3D model of inner/outer walls and plaque objects

The proposed methodοlogy includes 6 stages: in the first stage the CTA images are pre-processed to remove the artifacts and detect the vessel silhouette. In the second stage the artery borders are roughly defined and used to extract the initial centerline of the vessel. In the third stage, the plaque parameters are adapted in the CTA's HU scale to optimize a 4-component Gaussian Mixture Model (GMM) [22] to the HU histogram. In the fourth stage, radial images are produced perpendicular by to the initial centerline which is further processed in the fifth stage where the lumen and the outer wall border of the vessel are detected and the plaque region is estimated. Finally, in the sixth stage the 3D surfaces of the lumen - outer vessel wall and CP plaques are constructed. The stages of the proposed methodology are shown schematically in Fig. 1.

### Preprocessing

A preprocessing stage is applied on the acquired CTA images which includes:

- contrast enhancement [23], which maps the intensity values of the image to new values such that, 1 % of the data is saturated at low and high intensities of the input data,
- image thresholding [24], which allows identification of the vessel [25],
- and the application of a Frangi Vesselness filter (using the default parameters suggested by Frangi et al.) [26] which permits detection of structures which correspond to coronary vessels.

### Rough artery border detection

The following process is implemented to extract the initial lumen borders and define a first approximation of the luminal centerline:

- The user defines a seed point in the main artery (the only user action in the whole methodology).
- A region growing method is applied using 26-connected pixels neighbor connectivity for lumen border detection.
- Edge Detection is applied to estimate the lumen borders.
- The lumen border are used to define an initial vessel centerline using the center of gravity of each curve [27]. Then the centerline is smoothed (using moving average filter) in order to have a smooth first derivative of the centerline curve.

**Fig. 2:**
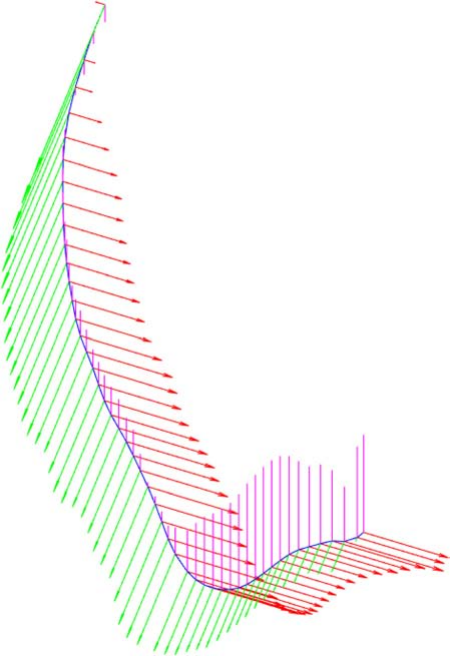
The centerline (blue) of an artery segment with the derivatives (red, purple and green) at each point

The initial centerline points are fitted using B-Splines. B-Spline is used to provide smooth first derivatives needed for producing a radial image perpendicular to a specific centerline point (Fig. 2).

### Image HU modeling

The distribution of HU intensity over the artery is modeled by a Gaussian Mixture Model (GMM) [22]. A GMM model is a parametric probability density function which is represented as a weighted sum of *M* Gaussian component densities given as:

1 $$ p\left(x\Big|\lambda \right)={\displaystyle \sum_{i=1,\;..M}{w}_iN\left(x\Big|{\mu}_i,{\sigma}_i\right)}, $$

where *x* is a 1-dimensional continuous data (HU value), *w*_*i*_ are the mixture weights, *μ*_*i*_ and *σ*_*i*_ are the mean vector and the standard deviation of the component Gaussian densities (*N(x|μ*_*i*_*,σ*_*i*_*)*), respectively and *λ* is the set:

2 $$ \lambda =\left\{{w}_i,\kern0.24em {\mu}_i,\kern0.24em {\sigma}_i\right\} $$

The maximum posterior probability of the sample *x* to belong to a specific component *k*, is given as:

3 $$ P\left(M=k\Big|x\right)=\frac{w_kN\left(x;{\mu}_k,{\sigma}_k\right)}{{\displaystyle \sum_{i=1..M}{w}_iN\left(x;{\mu}_i,{\sigma}_i\right)}}. $$

In our approach each component of the GMM models corresponds to one of the following classes: i) lumen, ii) calcified plaque, iii) non-calcified plaque and iv) background. In order to train the GMM model the user manually selects 4 points that correspond to the four classes ({*X*_*L*_*,X*_*CP*_*,X*_*NCP*_,X_B_}) and then each pixel is classified to one of the four classes based on the class/component with the maximum posterior probability. For each selected point, 5 random points are automatically detected in a circular neighborhood of radius 1.

### Radial Image Projection (RIP)

**Fig. 3:**
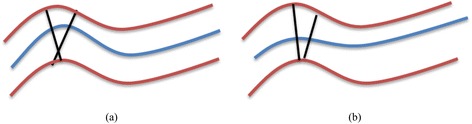
Centerline smoothing: **a** rather accurate centerline and the intersection imposed on perpendicular surfaces and **b** smoothed centerline where no intersections of perpendicular surfaces occurs

In the initially extracted centerline which is sampled every 0.2 mm, a point is defined and a radial image is produced perpendicularly to the centerline point using the B-spline derivatives extracted on the specific point [27]. These images are called Radial Intensity Projection (RIP) images. For creating the RIP images we used the 3D derivative vector of the b-spline corresponding to the centerline. On this way the continuity of the RIP images is achieved. For an accurate RIP image the estimated centerline point is required to lie in the middle of the lumen. However, as described in Section II-B the centerline is smoothed in order to avoid surface intersections as it is depicted in Fig. 3. This smoothing results in an offset of the estimated centerline from lumen’s center. In order to overcome this problem an iterative correction process is applied. The iterative process includes the following steps:

- The two bullets should be before Fig. 3. The radial image is produced for the given centerline and the current offset (initially the offset is set to zero).
- The center of mass of the radial image is calculated and a new offset is computed.

**Fig. 4:**
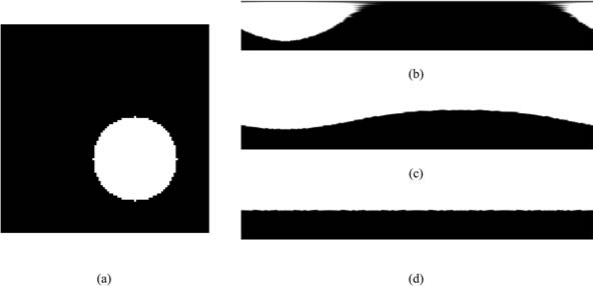
The radial image correction process on an artificial image **a** with large offset from the center, **b** in the first iteration, **c** in the third iteration and **d** in the last (fifth) iteration

**Fig. 5:**
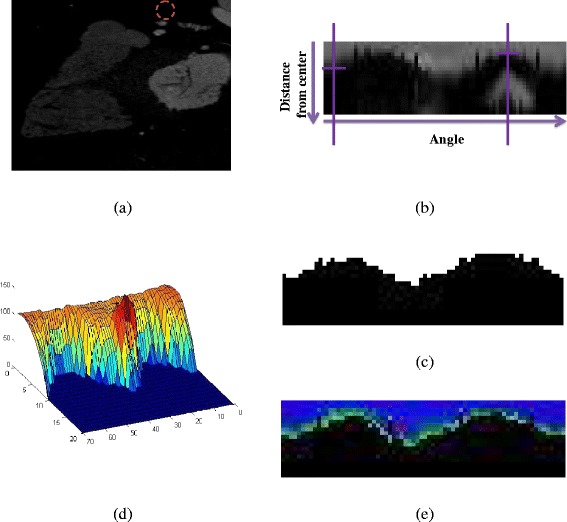
**a** The original DICOM image. In the red circle is the vessel region detected based on the initial stages of our methodology (stages 1, 2), **b** The radial image produced taking the RIP image corresponding to the centerline point of the specific vessel region and zero crosses of the RIP image, **c** The resulting ROI, **d** The HU profile from the RIP image and the extracted ROI, and **e** The classified ROI

The process is repeated five times (2, 3, 4 and 6 repetitions were also tested), number which is sufficient to minimize the offset. An example of the above procedure is depicted in Fig. 4 for a synthetic image with a binary circle with an initial large offset from the center (Fig. 4-(a)). The initial center of the circle should lie in the center of the image. It can be observed that after a few iterations the radial image is corrected (Fig. 4-(d)). After the correction step the final RIP image is used for the object extraction process. An example of the RIP image is given in Fig. 5.

### Inner - outer vessel wall and plaque region detection

From the RIP of each slice we extract the Region of Interest (ROI) of the artery. The ROI is extracted based on the detection of the zero crosses (HU < −130) [25], (Fig. 5-b). The areas below the zero crossing are removed (Fig. 5-c). After defining the ROI, the HU profile of the RIP image is calculated for each radius interval (Fig. 5-d). Based on the profile, we classify the ROI using the GMM model (Fig. 5-e).

Based on the ROI classification we define profile regions. The lumen wall is set to the first non-lumen point. The outer vessel wall is set to the last non-background point. Therefore, two curves are created one for the lumen and one for the outer vessel wall. After smoothing, the curves are transformed back to the Cartesian coordinates.

**Fig. 6:**
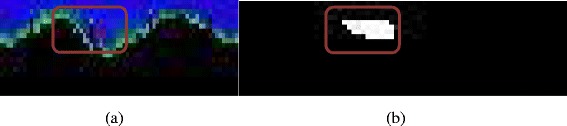
**a** Region with pixels classified as calcific plaque, and **b** the corresponding thresholded image where calcified objects are detected

Using the original radial image, the area between the inner and outer vessel wall defines the plaque region. The classified ROI (Fig. 6-(a)) is thresholded (the threshold is 0.5) according to the GMM probability estimations, keeping only pixels belonging to the specific plaque type (Fig. 6-(b)) resulting to binary objects. Then these binary objects are identified and the boundary of those objects is extracted. Smoothing is performed on the extracted boundary curves. Based on the physical size of each pixel the area of the plaque can be determined.

### 3D Surface construction

*N* points for each of the inner/outer vessel wall and plaque lesions are extracted clock wisely for each image of the centerline. Then a triangulation approach is implemented to construct the mesh surfaces. The border points (*N*) (inner/outer wall or plaque) of two sequential images, frame *i* and frame *i + 1* are connected constructing a triangle mesh. Thus 2*xN* surfaces are constructed for each pair of sequential images.

### Dataset

The data acquired from 20 patients, that underwent coronary angiography, IVUS imaging and CTA for clinical purposes, were used to validate the proposed methodology. Data collection were provided by the Invasive Cardiology Laboratory of the IFC-CNR, Pisa and by Heart Institute, University of Sao Paulo, São Paulo, Brazil. The study protocols were approved by the local Ethics Committees (IFC-CNR data: Comitato Etico, Azienda Ospedaliero-Universitaria Pisa, Pisa, Italy and Sao Paulo Data: Ethics Committee of the Clinics Hospital of the University of Sao Paulo Medical School - CAPPesq, Hospital das Clínicas da Faculdade de Medicina da U S P, Sao Paolo, Brazil) and written informed consent for participation in the study was obtained from participants.

### IFC-CNR data

Coronary CTA was performed using a 64-slice scanner (Light Speed, General Electric Health Care). Eight patients with a significant (>50 %) luminal stenosis on CTA had further invasive investigations. Coronary angiography was performed with the use of a flat panel system (Innova 2100, GEHC). To further assess the severity of the detected lesions on X-ray angiography, IVUS imaging was performed with the use of 64-crystal electronic ultrasound probe (Eagle-Eye, Volcano corporation) that was pulled-back by an automated pull back device at a speed 0.5 mm/sec. The acquired IVUS and CTA data were stored in DICOM format and transferred to a workstation for off-line analysis.

### Sao Paulo data

Twelve patients were enrolled in the study and had a clinical indication for CTA according to the guidelines and significant (>50 %) luminal stenosis was confirmed by CTA. The patients were further evaluated for coronary lesions using IVUS. The IVUS examination was performed using a 20 MHz electronic multi-array 2.9 F catheter (Eagle Eye^®^, Volcano Corporation Inc) connected to a dedicated console (InVision Gold^®^, Volcano Corporation Inc., San Diego, CA, USA). Acquisition was performed during motorized pullback at a constant speed of 0.5 mm/s (R-100^®^ pullback device, Volcano Corporation Inc., San Diego, Ca, USA). Patients with a heart rate greater than 65 bpm received up to 15 mg of intravenous metoprolol before the acquisition of MDCT images, unless contra-indicated. Sublingual nitrate was given to all patients prior to the acquisition of MDCT images. All scans were performed utilizing a 64-slice MDCT scanner (Aquillion 64TM, Toshiba Medical Systems, Japan). After the intravenous injection of contrast media (Iopamidol, 370 mg iodine/ml, Bracco), the helical scan for CT coronary angiography was triggered once a threshold of 180 Hounsfield units (HU) was reached at the descending aorta.

All CTA (eight IFC-CNR and twelve Sao Paulo) data were processed using the proposed methodology and their corresponding IVUS data (eight IFC-CNR and twelve Sao Paulo) using a previously described plaque characterization method [21]. CP was detected in 20 IVUS examinations and were used to compare the 3D CP detected by the proposed CTA methodology.

### CT-IVUS comparison

To compare the 3D reconstructed arteries and the detected 3D CP detected by the proposed methodology, IVUS plaque characterization [21] and 3D IVUS reconstruction [28] was performed in corresponding segments and the non-invasive-based (CTA) and invasive imaging (IVUS) based models were compared. Experts selected manually, segments of arteries which were assessed with both CTA and IVUS imaging. Side branches were used as fiducial points to define correspondence between CTA and IVUS.

### Artery and CP reconstruction using IVUS

IVUS based reconstruction was performed using the methodology introduced in [28]. The method combines IVUS and X-ray angiography and places the lumen and media adventitia borders detected in IVUS frames [21] onto the 3D luminal centerline derived from two angiographic projections. In the present work instead of using the 3D centerline derived from angiography, we used the 3D center line derived from CTA. To detect the CP in the selected IVUS segments a recently developed plaque characterization method [21] was employed. Hence, the lumen-outer vessel wall morphology and the CP of the arteries are reconstructed by combining IVUS and CTA.

### Comparison metrics

**Fig. 7:**
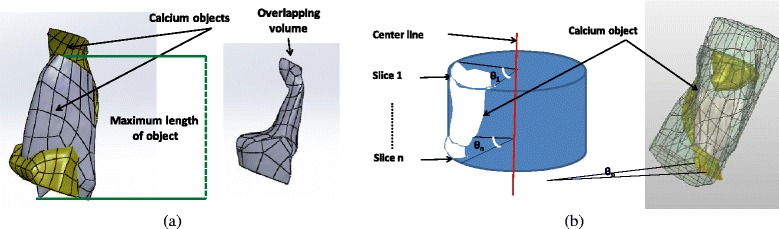
**a** The maximum length of a CTA object and the overlapping volume area with the IVUS object, and **b** schematic presentation of the inner angle metric used to compare the two objects

The inner and outer walls reconstructed by the two modalities were compared using their volume and surface area. The 3D CP objects detected by the two modalities were compared using the following metrics: volume, surface area (the total area of the object's faces), maximum length, inner angle and the overlapping volume (volume detected in both CTA and IVUS models) between two objects. As maximum length of an object we denote the distance between the first and the last voxel of the object along the centerline and as inner angle *θ*_*In*_, we denote the mean of the angles that define the circumferential extent of the object (Fig. 7). For an object having *n* 2D slices and angle *θ* angles, the *θ*_*In*_ is defined as:

4 $$ {\theta}_{In}=\frac{{\displaystyle \sum_{i=1}^n{\theta}_i}}{n} $$

## Results

### Sensitivity analysis of GMM initialization

To examine the sensitivity of the GMM model for each set of HU units (selected by the user): *X ∈ {X*_*L*_*,X*_*CP*_*,X*_*NCP*_*,X*_*B*_} a new set is calculated: *X*^'^ ∈ {*X*_*L*_^'^, *X*_*CP*_^'^, *X*_*NCP*_^'^, *X*_*B*_^'^}, where *X*^'^ = *X* ± *ΔΤ* (∆T was randomly added or subtracted from *X* with probability 0.5), ∆T = (20, 40, 60, 80, 100, 120, 140, 160, 180, 200). Therefore, for each *X*^'^ the GMM parameters are estimated and produce the inner and outer volume of three arteries that were used for testing the sensitivity of GMM initialization. To estimate the percentage of volume change (*Volume*^*ΔΤ*^) for each ∆T (or *X*^'^) we computed the percentage change as:

5 $$ C=100-\frac{\left| Volum{e}^{\varDelta T}- Volum{e}^0\right|}{Volum{e}^0}, $$

where *Volume*^0^ is the initial volume. However, the computed volumes in all three arteries which were used to test the sensitivity of GMM initialization, did not change significantly. In all three arteries, both inner and outer volumes, *C* ranged from 99.4 - 99.9 %.

### CT-IVUS

**Fig. 8:**
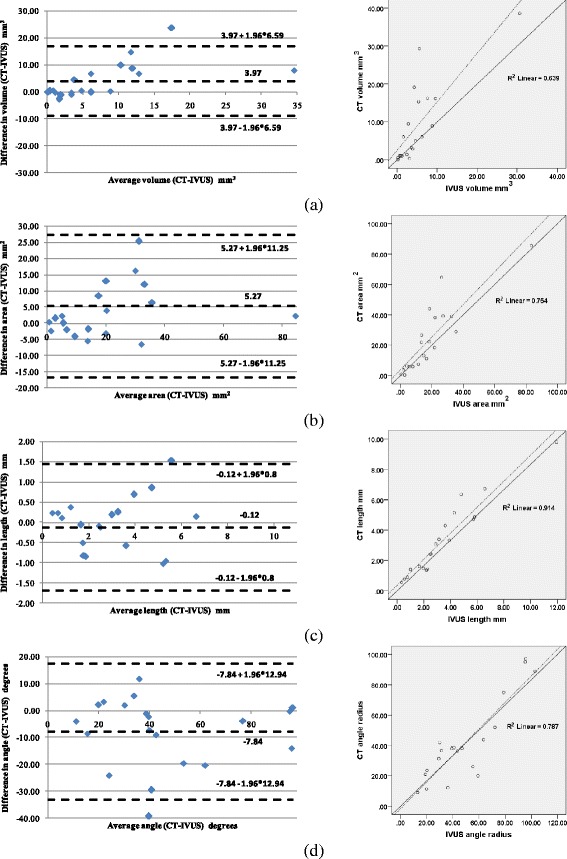
Bland-Altman (left) and correlation plots (right) for CTA-IVUS 3D objects comparison: **a** Volume comparison, **b** Surface area comparison, **c** Maximum length comparison, and **d** Inner angle comparison. Correlation plots: the solid line is the line of identity, while the dashed line is the regression line

**Fig. 9:**
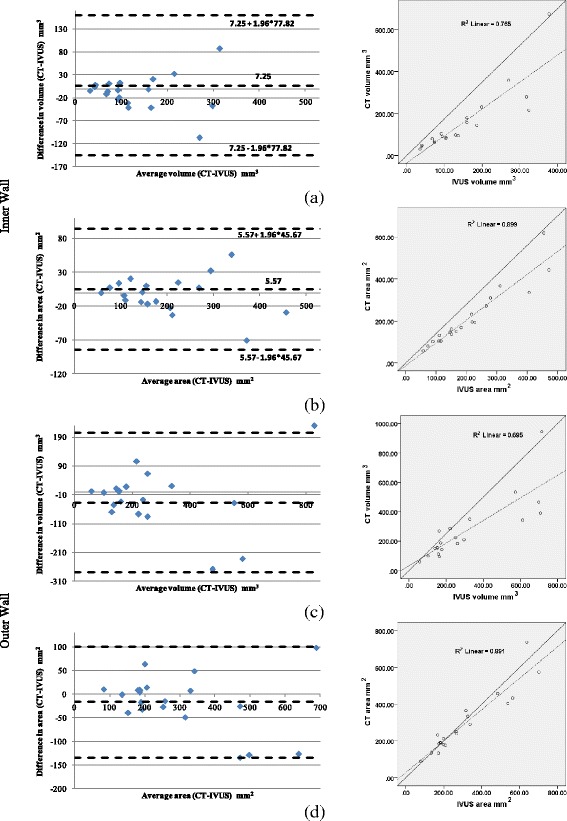
Bland-Altman (left) and correlation plots (right) for CTA and IVUS for the: **a** lumen volume, **b** Lumen surface area, **c** Outer vessel wall volume, **d** Outer vessel surface area. Correlation plots: the solid line is the line of identity, while the dashed line is the regression line

**Fig. 10:**
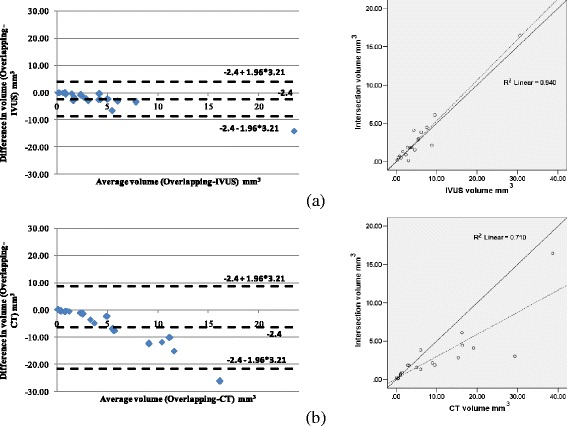
Bland-Altman (left) and correlation plots (right) for Volume intersection 3D objects comparison: **a** Intersection vs IVUS volume comparison, and **b** Intersection vs CT volume comparison. Correlation plots: the solid line is the line of identity, while the dashed line is the regression line

Using the previously described metrics, Pearson Correlation Coefficient (Table 1) and Bland–Altman plots were computed to measure the correlation between CTA and IVUS CP, inner and outer wall. The Bland-Altman plots and correlation plots results for detecting CP and inner outer wall are shown in Fig. 8 and Fig. 9, respectively. Fig. 10 presents the Bland-Altman and correlation plot results for the overlapping volume of CP objects when compared to the IVUS or to CT CP volumes.

**Table 1:** CTA and IVUS comparison of volume, surface area, length and maximum inner angle

	CP objects		Inner wall		Outer wall	
Metrics	Pearson's correlation r	Degree of correlation R^2^	Pearson's correlation r	Degree of correlation R^2^	Pearson's correlation r	Degree of correlation R^2^
Volume	0.79	0.64	0.87	0.76	0.83	0.69
Volume Intersection vs IVUS	0.96	0.94	-	-	-	-
Volume Intersection vs CT	0.82	0.71	-	-	-	-
Surface area	0.86	0.75	0.94	0.89	0.94	0.89
Length	0.95	0.91	-	-	-	-
Angle	0.88	0.78	-	-	-	-

**Fig. 11:**
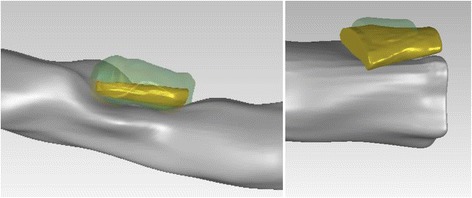
Lumen and 3D CP objects detected by CTA and IVUS: the gray object corresponds to lumen, the gold object to CPs detected by IVUS and the transparent green object correspond to CP detected by CTA. The volume mismatching between the objects is increased as the distance from the lumen to the plaque voxels is increased

The volume comparison results showed that 3D CP plaques, and the inner and outer vessel wall volumes of CTA correlated well with those obtained from IVUS, having Pearson's correlation (*r*) 0.79, 0.87 and 0.83, respectively. The correlation of CP objects is higher when their length (*r* = 0.86), inner angle (*r* = 0.95) and surface area (*r* = 0.88) is compared; meaning that although the methodology detects the CP objects in the same location within the arterial wall as they have the same maximum inner angle and length, their volume is increased in the CTA probably because IVUS is unable to assess then entire CP since there is an acoustic shadow behind calcium deposits [29, 30]. Another explanation of this finding is that in CTA high-attenuation structures, such as calcified plaques, appear enlarged (bloomed) [31]. These combined effects result in a moderate volume correlation (*r* = 0.79).The differences in the estimations of CTA and IVUS for the calcium component are shown in Fig. 11.

### Implementation

The CT segmentation method was developed in C/C++. The VTK library was used for 3D rendering and the comparison of the results was performed in Matlab. The time complexity of the proposed methodology is less than 15 seconds for an arterial segment of 20 mm length using a desktop computer with an Intel core i7 processor and 16 GB of RAM, while manual component selection takes less than 30 sec for an experienced user.

## Discussion

In this work, we presented a semi-automated methodology for reconstructing the lumen, outer vessel wall and CP plaque in arteries using CTA images. The methodology is based on a 3-component GMM [32] to detect the lumen, outer vessel wall borders and the CP and NCP plaque components and it is the only semi-automated methodology available in the literature that enables 3D reconstruction of coronary anatomy and plaque characterization. The results of the proposed approach demonstrated that our methodology provides geometrically correct 3D models and permits reliable characterization of the composition of the plaque.

Over the last years several methodologies have been designed for the segmentation of the CTA cross sectional images and the characterization of plaque components [8, 15–17, 19, 20]. However, these methodologies are time consuming since they often require corrections of the detected borders and none of them is capable to reconstruct the coronary anatomy in an automated fashion allowing comprehensive visualization of luminal and outer vessel wall morphology, and assessment of the distribution of different plaque components. The proposed approach overcomes the abovementioned limitations as it permits fast semi-automated segmentation and tissue characterization, reliable coronary reconstruction and 3D representation of coronary anatomy and pathology.

In contrast to previous works which focus on a simplistic comparison of the volumes and areas of the regions of interest in CTA and IVUS, we implemented a more complex comparison methodology that allows assessment of the 3D reconstructed models. We found high correlation between IVUS and CTA models for the lumen, and outer vessel wall surfaces and volumes. We also demonstrated that the CP have the same circumferential and longitudinal extent, however, there was a relative small difference in the computed volumes (*r* = 0.79); fact that should be attributed to the fact that IVUS cannot portray the entire CP (acoustic shadow) [29, 30] and to that in CTA large CP plaques appear enlarged (blooming effect) [31]. Table 2 presents a comparison between the results of the proposed methodology and the methodologies proposed in the literature. Although, other studies [8, 16] demonstrated a higher volume correlation (Table 2) for the CP between the two imaging modalities, they have not compared the exact location of the CP objects (overlapping volume, Fig. 10). A high correlation was noted (*r* = 0.94) when we compared the CP overlapping volume of the IVUS and CT derived reconstructions, while a moderate correlation (*r* = 0.71) was noted when compared the CP overlapping volume between CT and IVUS. This additional comparison step demonstrates that the CP objects derived by the two imaging modalities are located in the same position.

**Table 2:** Comparison of the proposed method with respect to CP detection with the literature

Method	Degree of correlation R^2^	Pearson's correlation r	Note
Brodoefel et al.[17]	0.07	0.1	-
Hur et al. [19]	-	0.7	Only area comparison
Papadopoulou et al. [8]	-	0.91	Calcium is merged with Mixed plaque
Utsunomiya et al. [20]	-	0.40	Only area comparison
Voros et al.[16]	-	0.84	-
de Graaf et al. [18]	-	0.73	-
Proposed method	0.64	0.79	-

The proposed methodology utilizes CT data from two different scanners. This is due to the fact that the availability of combined CT-IVUS data is limited and thus, in order to acquire an adequate number of cases for the analysis, data from different institutions and subsequently different equipment were used. However, the usage of such different data shows the applicability of the methodology in different settings and different clinical environments. Moreover, the proposed methodology is anticipated to enhance the applications of CTA in the study of atherosclerosis as it allows expedite coronary reconstruction and comprehensive representation of the coronary anatomy and plaque distribution. Thus, the obtained 3D models are expected to allow blood flow simulation and evaluation of the shear stress distribution and assessment of the impact of flow dynamics on atherosclerotic disease progression.

The presented methodology is based on modeling the distribution of HU intensity over the artery using a GMM model. The GMM model is trained using manually selected points which correspond to four classes: lumen, calcified plaque, non-calcified plaque and background. Nowadays new Radiation Reduction Techniques (RRT) have been developed and embedded in CTA scanners (i.e. CARE kV Siemens Healthcare). RRT automatically optimizes the medium contrast dose and the X-ray tube voltage for each individual [33]. A significant reduction on the CTA tube voltage (up ton100 kV) is associated with a significant reduction in radiation exposure [34], a lower dosage of medium contrast and higher contrast enhancement in the produced CTA images [35]. Changing the tube voltage of the CTA scanner, the HU values change as well. Therefore, the expected change in HU values by a possible tube voltage reduction may affect the manually point selection of inexperienced users. Although, a significant reduction of the tube voltage (up to 100 kV) will increase the image noise as well as contrast-to-noise ratio, it will not impair diagnostic image quality [34]. On the contrary, when reducing the tube voltage (up to 100 kV) we have an increment of the blooming artifacts created from dense structures (stents) which may preclude an accurate lumen detection [36]. In all CTA scanners the blooming effect results from beam hardening and causes the stent struts to overlap the vessel lumen. Currently, when the methodology is applied in stented segments erroneous CP objects are detected due to stent's blooming effect. Therefore, circular CP objects detected by the method are considered as stents and these segments are not included in the final outcome. Similarly, the results of bifurcated segments are not included in the final outcome as the reconstruction of coronary bifurcations is a more complex process. However, in order to investigate the use of the proposed methodology in the new RRT CTA scanners a comparative study should be implemented using data derived from RRT CTA scanners, data derived from non-RRT CTA scanners and histological data. Such a study will allow the adaptation of the proposed methodology and its application in RRT CTA scanners and will enhance the reliability of the results as a direct comparison to the gold standard (histology) will be available.

## Conclusions

We developed a semi-automated methodology for the segmentation of the regions of interest and characterization of the composition of the atheroma in CTA images. The proposed approach allows coronary 3D reconstruction in almost real time and a holistic assessment of 3D plaque morphology and distribution. The proposed approach was extensively compared to IVUS findings using various comparison metrics. The results indicated that the proposed methodology is fast and accurate and thus it is likely in the future to have applications in research and clinical arena.

## Abbreviations

3D: Three-dimensional
IVUS: Intravascular ultrasound
CTA: Computed tomography angiography
CP: Calcified plaque
NCP: Non-calcified plaque
VH: Virtual histology
HU: Hounsfield units
GMM: Gaussian mixture model
RIP: Radial image projection
ROI: Region of interest
